# An evaluation of the challenges to developing tumor BRCA1 and BRCA2 testing methodologies for clinical practice

**DOI:** 10.1002/humu.23375

**Published:** 2017-12-28

**Authors:** Gillian Ellison, Miika Ahdesmäki, Sally Luke, Paul M. Waring, Andrew Wallace, Ronnie Wright, Benno Röthlisberger, Katja Ludin, Sabine Merkelbach‐Bruse, Carina Heydt, Marjolijn J.L. Ligtenberg, Arjen R. Mensenkamp, David Gonzalez de Castro, Thomas Jones, Ana Vivancos, Olga Kondrashova, Patrick Pauwels, Christine Weyn, Eric Hahnen, Jan Hauke, Richie Soong, Zhongwu Lai, Brian Dougherty, T. Hedley Carr, Justin Johnson, John Mills, J. Carl Barrett

**Affiliations:** ^1^ Precision Medicine and Genomics IMED Biotech Unit AstraZeneca Macclesfield UK; ^2^ Translational Science, Oncology IMED Biotech Unit AstraZeneca Cambridge UK; ^3^ R&D Information AstraZeneca Cambridge UK; ^4^ Department of Pathology University of Melbourne Parkville Melbourne Victoria Australia; ^5^ Genomic Diagnostics Laboratory, Manchester Centre for Genomic Medicine Central Manchester University Hospitals NHS Foundation Trust, Saint Mary's Hospital Manchester UK; ^6^ Kantonsspital Aarau Institut für Labormedizin, Abteilung für Medizinische Genetik Aarau Switzerland; ^7^ Institute of Pathology University Hospital Cologne Cologne Germany; ^8^ Department of Human Genetics Radboud University Medical Center Nijmegen The Netherlands; ^9^ Department of Pathology Radboud University Medical Center Nijmegen The Netherlands; ^10^ Centre for Cancer Research and Cell Biology Queen's University Belfast Belfast UK; ^11^ The Centre for Molecular Pathology The Royal Marsden NHS FT Sutton UK; ^12^ Laboratory 2.01 Vall d'Hebron Institute of Oncology (VHIO) Barcelona Spain; ^13^ Center for Oncological Research (CORE), Pathology Department University Hospital Antwerp (UZA) Edegem Belgium; ^14^ Center for Hereditary Breast and Ovarian Cancer and Center for Integrated Oncology (CIO) Medical Faculty, University Hospital Cologne Cologne Germany; ^15^ Cancer Science Institute of Singapore, and Department of Pathology National University of Singapore Singapore Singapore; ^16^ Bioscience, Oncology, IMED Biotech Unit AstraZeneca, IMED Oncology Waltham Massachusetts; ^17^ Translational Science, Oncology, IMED Biotech Unit AstraZeneca Waltham Massachusetts

**Keywords:** diagnostic, FFPE, NGS, PARP, t*BRCA*

## Abstract

Ovarian cancer patients with germline or somatic pathogenic variants benefit from treatment with poly ADP ribose polymerase (PARP) inhibitors. Tumor *BRCA1/2* testing is more challenging than germline testing as the majority of samples are formalin‐fixed paraffin embedded (FFPE), the tumor genome is complex, and the allelic fraction of somatic variants can be low. We collaborated with 10 laboratories testing *BRCA1/2* in tumors to compare different approaches to identify clinically important variants within FFPE tumor DNA samples. This was not a proficiency study but an inter‐laboratory comparison to identify common issues. Each laboratory received the same tumor DNA samples ranging in genotype, quantity, quality, and variant allele frequency (VAF). Each laboratory performed their preferred next‐generation sequencing method to report on the variants. No false positive results were reported in this small study and the majority of methods detected the low VAF variants. A number of variants were not detected due to the bioinformatics analysis, variant classification, or insufficient DNA. The use of hybridization capture or short amplicon methods are recommended based on a bioinformatic assessment of the data. The study highlights the importance of establishing standards and standardization for t*BRCA* testing particularly when the test results dictate clinical decisions regarding life extending therapies.

**Abbreviations**
FFPE: formalin‐fixed paraffin‐embedded
LGR: large re‐arrangement
MLPA: multiplex ligation‐dependent probe amplification
NGS: next‐generation sequencing
PARP: poly ADP ribose polymerase
VUS: variant of uncertain significance

## 1 INTRODUCTION

Tumor *BRCA1* and *BRCA2* (t*BRCA1/2*) testing in ovarian cancer is of increasing clinical importance as ovarian cancer patients with both germline and somatic (only found in neoplastic cells) pathogenic *BRCA1/2* variants have been shown to benefit from treatment with poly ADP ribose polymerase (PARP) inhibitors (Ledermann et al., 2014; Lheureux et al., 2017). Somatic *BRCA1/2* pathogenic variants are found to be present in up to 7% of ovarian cancers in the first line or platinum‐sensitive relapsed clinical setting (Alsop et al., 2012; Dann et al., 2012; Hennessy et al., 2010; McAlpine et al., 2012; Merajver et al., 1995; Yang et al., 2011). This represents a significant population of women who could benefit from PARP inhibitors and about a third of all *BRCA* mutated patients in this setting. From a biological rationale perspective, it is envisaged that PARP inhibitors are active irrespective of whether a *BRCA1/2* variant is of germline or somatic origin as both result in the loss of function of both copies of *BRCA1* or *BRCA2* in the tumor (Dougherty et al., 2017). As *BRCA1/2* testing is now required to support treatment decisions in many countries, it is essential that testing is robust.

To identify patients with somatic *BRCA1/2* variants, the DNA from the tumor sample has to be analyzed. This is more technically challenging than germline testing, but does have the advantage that germline and somatic variants can be identified in a single sample taking the combined tumor *BRCA1/2* mutation frequency to almost a third of high grade serous ovarian cancers (Pennington et al., 2014). The majority of clinical tumor samples have been formalin fixed and paraffin embedded (FFPE), resulting in technical challenges for both germline and somatic mutation testing. The tissue fixation process causes fragmentation and chemical modification to the DNA, leading, respectively, to PCR amplification failures and false positive sequencing results. Care must be taken to avoid misinterpreting sequencing artifacts (Ellison et al., 2010, 2015). The yields of amplifiable DNA tend to be much lower compared with DNA extracted from blood or fresh frozen tissue and can be a limiting factor when the entire coding region of two large, complex genes, such as *BRCA1* and *BRCA2*, need to be screened (the combined coding regions account for approximately 15 kb).

Moreover, for the detection of somatic variants, a low proportion of neoplastic cells compared with non‐neoplastic cells within the tumor can result in false negatives. Accordingly, methods established for routine germline *BRCA1/2* testing may not be suitable for tumor testing as they are not optimized for highly fragmented DNA or to detect potentially low‐level somatic variants against a background of normal DNA. These issues limit the choice of methods suitable to robustly detect both germline and somatic *BRCA1/2* variants in tumor‐derived DNA, with next‐generation sequencing (NGS) currently being the best available option to conduct full gene screening.

Many clinical testing laboratories have now adopted NGS technologies for routine screening including germline *BRCA1/2* testing (Patton) and some diagnostics laboratories are beginning to apply this technology for tumor *BRCA1/2* (t*BRCA*) screening (Endris et al., 2016). NGS methods, equipment, data analysis, and experience are considerably variable across laboratories. To evaluate a range of tumor *BRCA1/2* testing approaches, we conducted a study with ten clinical laboratories to determine the ability of a spectrum of tumor *BRCA1/2* testing workflows to accurately identify t*BRCA* variants in clinical practice. A set of 12 FFPE tumor DNA samples with eight potentially clinically important variants (pathogenic, likely pathogenic, and variants of uncertain significance, VUS) were provided to all participating laboratories, including lower tumor variant allele frequency (VAF) somatic variants and varying amounts of DNA across the 12 samples (ranging between 64 and 443 ng; Table 1).

**Table 1:** DNA samples provided for testing

DNA sample	Variant	Clinical classification	DNA (ng/μl)	Total (ng)
1	*BRCA2* c.7007 + 1G > C	Pathogenic	12.7	443
2	No pathogenic variant		12.5	312
3	No pathogenic variant		9	225
4	*BRCA1* c.4675G > A p.(Glu1559Lys)	Pathogenic	5.3	186
5	*BRCA1* c.213‐11T > G	Pathogenic (known germline)	3.7	129
6	*BRCA1* c.1105delG p.(Asp369MetfsTer5)	Pathogenic	3.5	121
7	*BRCA1* exon13ins6kb	Pathogenic (known germline)	3.3	81
8	No pathogenic variant		2.6	64
9	*BRCA2* c.7788delAinsGGGT p.(Gly2596dup)	VUS	2.1	84
10	No pathogenic variant		1.9	68
11	*BRCA2* c.6952C > T p.(Arg2318Ter)–Admix ∼5%	Pathogenic (known germline)	1.2	72
12	*BRCA2* c.10024G > A p.(Glu3342Lys)–Admix ∼40%	VUS	1.1	66

Mutations and variants are named according to HGVS guidelines on mutation nomenclature (https://www.hgvs.org/mutnomen) using reference sequences *BRCA1* LRG_292t1 and *BRCA2* LRG_293t1.

Detecting copy number variation, that is, the duplication or deletion of DNA segments larger than 1 kb, in FFPE is a challenge especially when looking for single gene losses or gains (Jacobs et al., 2007; Michels et al., 2007). These copy number variants, also known as large re‐arrangements (LGRs), vary considerably in their frequency in different populations, ranging from less than 1% to greater than 20% for populations with a strong founder effect (Ewald et al., 2009). If tumor DNA is to be screened instead of a blood sample for germline testing only, it is important that this class of variant can be detected. Although only one participating laboratory used a method to detect large insertion or deletion variants, a variant of this category was included to allow us to evaluate the feasibility of detecting copy number changes in NGS data.

The ultimate purpose of the study was to highlight the importance of standards and standardization particularly when the test results dictate clinical decisions regarding therapies. This is analogous to the important lessons learned about HER2 testing from such studies that eventually led the ASCO/CAP to develop and implement guidelines for HER2 testing. No health economic assessment was carried out.

## 2 MATERIALS AND METHODS

### 2.1 Laboratory selection

Clinical diagnostic laboratories with an established tumor *BRCA1/2* testing process were invited to join the study after participating in an advisory meeting on tumor *BRCA1/2* testing in 2015. No other selection criteria were placed on participants. Of 12 invited participants, 10 laboratories able to complete the formal sample transfer authorization process joined the study, which also took place in 2015.

### 2.2 Preparation of DNA for test panel

Ovarian and breast tumor samples were obtained from Asterand (Detroit, MI) and collected with appropriate consents that had been reviewed and approved by relevant regulatory and ethical authorities (further details can be found at Asterand.com). The pathology data provided by the supplier were used to indicate suitability of the samples. No independent pathology review was conducted. Eight samples with a diverse but clinically representative range of *BRCA1/2* variants were selected for inclusion in the study (Table 1) as well as four *BRCA* wild‐type controls. The genotypes of these samples were known from previous *BRCA* screening and indicated all positive *tBRCA* variant samples had previously observed tumor variant allele frequencies of greater than 50% (Ellison et al., 2015) or were from patients with known *BRCA1/2* germline variants. For each sample, DNA was extracted from twenty 20 micron in total (two 20 micron sections per extraction) using the Qiamp DNA FFPE Tissue kit (Qiagen, Hilden, Germany) and pooled. The resulting DNA was quantified and assessed for quality by quantitative PCR using the 129 bp PCR amplicon from the human genomic DNA Quantification and QC Kit (KapaBiosystems, Wilmington, MA).

Two admixtures of *BRCA2* mutated FFPE DNA mixed with a non‐mutated FFPE DNA sample were made to mimic low‐level mutant samples that could be present in *BRCA* somatically mutated only tumors. The resulting test panel is described in Table 1. The DNA was divided into equal aliquots such that all laboratories received the same amount of DNA for a given sample. For some samples, this was less than the recommended DNA input for the laboratories’ established method however the participants were requested to analyze all samples to allow comparison over a range of conditions. One laboratory (P3) only received nine samples as there was insufficient DNA available.

All DNA samples were re‐analyzed by a commercial testing laboratory (Foundation Medicine, Cambridge, MA) using the Foundation One V.7, 394 gene panel (hybrid enrichment method) to verify the expected genotypes and to provide a reference dataset to be used in the event of discordance. At the time of writing this article, the Foundation Medicine test was the only FDA approved tissue test for *BRCA1/2*.

### 2.3 *BRCA1/2* sequencing and bioinformatics

Laboratories were asked to conduct the analyses using their t*BRCA1/2* NGS testing process (Table 2) and were asked to report any significant findings in addition to making available their sequence level data (binary sequence alignment [BAM] files or equivalent). The primary analysis was blinded. After the blinded analysis, any differences between the known genotype and that reported by the laboratory were revealed to the participating laboratory to enable them to re‐evaluate their data and determine, if possible, the reason for any apparent discrepancy.

**Table 2:** *BRCA1/2* tumor testing processes

Process	NGS process	Optimal DNA amount, by quantitative method	NGS instrument	Data analysis tools used
P1	*BRCA1/2* GeneRead Panel (QIAGEN)	80 ng by Q‐PCR	MiSeq	bwa, Varscan, dreep, pindel, Ensembl, ExAc, EVS, SIFT, Polyphen
P2	*BRCA1/2* GeneRead Panel (QIAGEN)	Not stated	MiSeq	Analysis performed by Sophia Genetics
P3	*BRCA1/2* GeneRead Panel GeneRead DNA Library I Core Kit, GeneRead DNA I Amp Kit (all QIAGEN), with modifications	40 ng by Q‐PCR	MiSeq	bwa, Blat, SAMtools
P4	Laboratory developed custom amplicon panel for *BRCA1/2* based on single molecule molecular inversion probes (smMIP) [22,23]	100 ng by Qubit	NextSeq 500	SeqPilot, SeqNext module
P5	TSCA 2‐gene HRD panel (Illumina)	150 ng by Qubit	MiSeq	MiSeq Reporter, Variant Studio and BaseSpace
P6	Laboratory developed custom amplicon panel for *BRCA1/2*, including use of NEBNext® kits	500 ng by nanodrop	MiSeq	bwa, SAMtools, Indel realign (GATK), VarScan2
P7	TruSeq custom HRD panel (30 genes) and MLPA NGS 12 gene panel [21]	>170 ng by Qubit	HiSeq	MiSeq Reporter, Variant studio, In house Amplivar pipeline
P8	*BRCA* Tumor MASTR Plus Kit (Multiplicom)	200 ng by Qubit	MiSeq	SeqNext software (JSI)
P9	TruRisk Sureselect XT (Agilent) 48 gene panel	200 ng by Qubit	MiSeq	In house pipeline, Varpipe 2.15
P10	TruSight Cancer sequencing 94 gene panel (Illumina)	150 ng Qubit	MiSeq	MiSeq Reporter, bwa, GATK, Variant Studio
Reference method	Foundation One V.7, 394 gene panel	200 ng Qubit	HiSeq 2500	Bwa, GATK, in‐house pipeline

The BAM files submitted by participant laboratories were re‐analyzed to help explain any sequencing differences. This was done using Bcbio 0.9.6 (Chapman) in order to realign the data using bwa (Li & Durbin, 2010) and perform further quality control (QC). Laboratories P5 and P8 supplied 2 BAM files per sample and P6 did not provide any BAM files. P8 used a protocol where two strands were analyzed separately and a variant was propagated further only if it was called in both strands. P5 performed the sequencing twice due to sample failures and provided sequencing data for both runs. The size of the BAM files ranged from 12 to 96 MB. For data derived from hybridization capture technologies, duplicates were marked using samblaster (Faust & Hall, 2014). Variant calling in the sequence data was performed using VarDict (Lai et al., 2016) and variant effects annotated by snpEff (Cingolani et al., 2012). Filtering of non‐cancer variants was performed as per VarDict best practice (Lai et al., 2016). The transcript variants used in the analyses were NM_007294.3 and NM_000059.3 for *BRCA1* and *BRCA2*, respectively.

Copy number analysis was performed using Seq2C (Lai) for exons and at the gene level for *BRCA1* and *BRCA2*. Log 2 values, normalized by sample median, were plotted for each exon.

## 3 RESULTS

The participating laboratories employed a range of methods and data analysis tools to screen for *BRCA1/2* variants in the DNA samples provided. The majority of laboratories (seven laboratories) used amplicons‐based NGS methods that only analyzed *BRCA1* and *BRCA2*, one laboratory (P7) used a combination of an amplicons‐based panel that screened 30 genes including *BRCA1/2* and a 12 gene multiplex ligation‐dependent probe amplification (MLPA) NGS assay (Kondrashova et al., 2015), and two laboratories (P9, P10) used hybridization capture‐based approaches that analyzed multiple genes (48 and 94 genes, respectively). The reference laboratory (Foundation Medicine) also used a hybridization capture‐based approach using their proprietary Foundation One V.7 panel of 394 genes. The majority of laboratories outsourced the design of their customized gene panels to commercial suppliers, but two laboratories used their own designs (P4, P6). Nine laboratories assembled their own bioinformatics pipelines using combinations of existing tools including custom developed tools in some cases. One laboratory (P2) used an external data analysis provider, Sophia Genetics, to analyze and interpret their data. The range of approaches used are summarized in Table 2.

**Figure 1:**
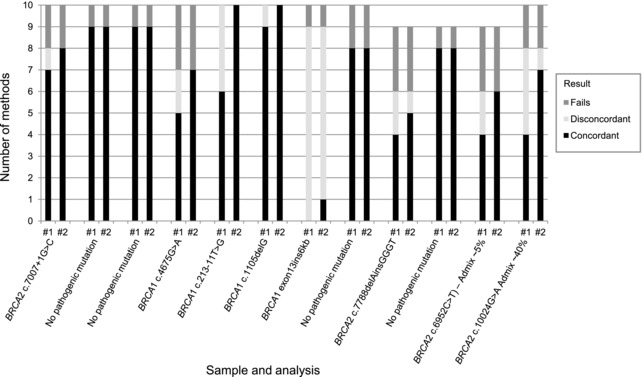
Summary of concordance of *BRCA1/2* genotyping results returned compared with expected genotypes after initial analysis and on re‐analysis. #1: Concordance of genotyping results returned, prior to knowledge of the expected result. #2: Concordance of genotyping results with knowledge of the expected result after re‐analysis. After initial analysis, the expected genotypes were found by at least four laboratories for all samples, except for the large re‐arrangement *BRCA1* exon13ins6kb sample, which would not have been detected by the analysis of sequencing level data. Re‐analysis revealed that 16 variants were present in the data that had not been reported in the initial analysis. Only three variants were not detected in the sequencing re‐analysis including *BRCA1* exon 13 insertion of 6 kb using all but one method. The additional low confidence variants are not included on this chart as they were likely not to be reported and therefore not considered to be a significant risk of error

The results returned by the participating laboratories were compared with the expected results from previous *BRCA1/2* screening of the samples or known germline *BRCA1/2* status of the patients. All the results from the primary sequencing analysis carried out by the participating laboratories are summarized in Table 3 and Figure 1.

**Table 3:** Summary of *BRCA1/2* genotyping results compared with expected genotypes after initial analysis and on re‐analysis

Process/Variant	Analysis	P1	P2	P3	P4	P5	P6	P7	P8	P9	P10	Concordance if data passed QC	Fails
Sample 1 *BRCA2* c.7007 + 1G > C	Initial analysis	C	C	C	C	C	F	C	F	C	DC	7/8 (88%)	2/10 (2%)
	Re‐evaluation	C	C	C	C	C	F	C	F	C	C 4	8/8 (100%)	2/10 (2%)
Sample 2 No pathogenic variant	Initial analysis	C	C	C	C	C	F	C	C	C	C	9/9 (100%)	1/10 (1%)
	Re‐evaluation	C	C	C	C	C	F	C	C	C	C	9/9 (100%)	1/10 (1%)
Sample 3 No pathogenic variant	Initial analysis	C	C	C	C	C	F	C	C	C	C	9/9 (100%)	1/10 (1%)
	Re‐evaluation	C	C	C	C	C	F	C	C	C	C	9/9 (100%)	1/10 (1%
Sample 4 *BRCA1* c.4675G > A	Initial analysis	C	C	C*	F	C	F	C	F	C	DC	6/7 (71%)	3/10 (30%)
* *	Re‐evaluation	C	C	C	F	C	F	C	F	C	C 4	7/7 (100%)	3/10 (30%)
Sample 5 *BRCA1* c.213‐11T > G	Initial analysis	C	C	DC	C	C	DC	C	DC	C	DC	6/10 (60%)	0/10 (0%)
* *	Re‐evaluation	C	C	C 1	C	C	C 1	C	C 1	C	C 1	10/10 (100%)	0/10 (0%)
Sample 6 *BRCA1* c.1105delG	Initial analysis	C	C	C	C	C	C	C	C	C	DC	9/10 (90%)	0/10 (0%)
* *	Re‐evaluation	C	C	C	C	C	C	C	C	C	C 4	10/10 (100%)	0/10 (0%)
Sample 7 *BRCA1* exon13ins6kb	Initial analysis	DC	DC	DC	DC	DC	DC	F	DC	DC	DC	0/9 (100%)	1/10 (10%)
* *	Re‐evaluation	DC	DC	DC	DC	DC	DC	F	DC	C	DC	1/9 (11%)	1/10 (10%)
Sample 8 No pathogenic variant	Initial analysis	C	C	C	C	F	C	F	C	C	C	8/8 (100%)	2/10 (2%)
	Re‐evaluation	C	C	C	C	F	C	F	C	C	C	8/8 (100%)	2/10 (2%)
Sample 9 *BRCA2* c.7788delAinsGGGT	Initial analysis	C	C	No DNA supplied	F	DC	F	F	C	C	DC	4/6 (67%)	3/9 (33%)
* *	Re‐evaluation	C	C		F	DC 5	F	F	C	C	C 4	5/6 (83%)	3/9 (33%)
Sample 10 No pathogenic variant	Initial analysis	C	C	No DNA supplied	C	C	C	F	C	C	C	8/8 (100%)	1/9 (11%)
	Re‐evaluation	C	C		C	C	C	F	C	C	C	8/8 (100%)	1/9 (11%)
Sample 11 *BRCA2* c.6952C > T– Admix ∼5%	Initial analysis	C	C	No DNA supplied	C	C	F	F	DC	F	DC	4/6 (67%)	3/9 (33%)
* *	Re‐evaluation	C	C		C	C	F	F	C 3	F	C 3	6/6 (100%)	3/9 (33%)
Sample 12 *BRCA2* c.10024G > A Admix ∼40%	Initial analysis	C*	C	C	C	DC	F	F	DC	DC	DC	4/8 (50%)	2/10 (20%)
	Re‐evaluation	C	C	C	C	C 2	F	F	C 2	DC 6	C	7/8 (88%)	2/10 (20%)
Concordance if data passed QC	Initial analysis	11/12 (92%)	11/12 (92%)	7/9 (78%)	9/10 (90%)	7/11 (64%)	3/5 (60%)	6/6 (100%)	6/10 (60%)	9/11 (82%)	4/12 (33%)	73/98 (74%)	
	Re‐evaluation	11/12 (92%)	11/12 (92%)	8/9 (89%)	9/10 (90%)	9/11 (82%)	4/5 (80%)	6/6 (100%)	9/10 (90%)	10/11 (91%)	11/12 (92%)	87/98 (89%)	
Fails	Both	0/12 (0%)	0/12 (0%)	0/9 (0%)	2/12 (16%)	1/12 (8%)	7/12 (58%)	6/12 (50%)	2/12 (16%)	1/12 (8%)	0/12 (0%)		19/117 (16%)

C, Concordant with expected result; DC, discordant—expected variant not detected; F, failed laboratories QC criteria; ^*^, additional low confidence variant detected. Subcategory reason for initial non‐concordance after re‐analysis: 1, intronic variant >2 bp into the intron not reported; 2, variant reported as benign in database therefore not reported; 3, low‐level variant below acceptance cut‐off for variant detection; 4, incorrect reference sequence used for reporting; 5, no replicate for confirmation; 6, sub‐optimal DNA input (too low); F, failed laboratories QC criteria; ^*^, additional low confidence variant detected; C, concordant; DC, discordant/not present; F, failed laboratories QC criteria; subcategory reason for initial non‐concordance after re‐analysis: 1, intronic variant >2 bp into the intron not reported; 2, variant reported as benign in database therefore not reported; 3, low‐level variant below acceptance cut‐off for variant detection; 4, incorrect reference sequence used for reporting; 5, no replicate for confirmation; 6, sub‐optimal DNA input (too low).

### 3.1 Analysis success rates

Four laboratories’ methods satisfactorily analyzed all the samples provided. These were the three laboratories using the GeneRead *BRCA1/2* panel (P1, P2, P3* [*laboratory received only nine samples]), and P10 using the TruSight Cancer sequencing 94 gene panel. All other methods failed at least one sample, the reasons for which are described in the following section.

### 3.2 Test fails

In total, 19 out of a possible total of 117 analyses (16%) failed to generate a result of acceptable quality as judged by the analyzing laboratory (Table 3).

The laboratory developed custom amplicon panel for *BRCA1/2* based on smMIP (Neveling et al., 2017; Weren et al., 2017) (P4) failed two samples due to excessive input DNA quantity. The DNA concentration information originally supplied with the samples was measured by quantitative PCR. Based on the provided concentration information, the required assay DNA input amount dictated all provided material to be used in the analysis of the lower input samples. However, the quantitative PCR method reflected a much lower DNA amount than Qubit, which had been used to optimize the assay. For higher DNA quantity samples it was possible to repeat the test, and when the DNA input was re‐measured using a Qubit instrument, the test passed the laboratory's QC process. However, there was insufficient DNA to repeat two samples resulting in two test fails.

The TSCA‐2 gene HRD panel used by P5 failed one sample (sample 8) due to there being significantly less DNA than was recommended for optimal assay performance.

The laboratory developed custom amplicon panel for *BRCA1/2* used by P6 failed using both high (samples 1 through 4) and low (samples 9, 11, and 12) input DNA amounts, with only five samples in the mid‐range (as measured by the laboratory) of DNA supplied passing quality acceptance criteria for the assay.

The TruSeq custom 30 gene panel and 12 gene MLPA NGS assay used by P7 failed internal sample QC for six samples with DNA concentrations below that required for optimal performance of the assays.

The *BRCA* Tumor MASTR Plus Kit used by P8 failed in two of the higher DNA concentration samples but the reason for this was unknown.

The TruRisk^®^ SureSelect method used by P9 failed one of the lower input DNA samples, however hybrid capture methods tend to require higher DNA input for optimal performance so low DNA is the likely reason for failure.

### 3.3 Discordant sequencing results

Out of a total of 98 analyses that passed internal laboratory quality assessment, 25 instances of potentially clinically important (pathogenic, likely pathogenic, and VUS) variants were not reported (Table 3) and there were two additional variants of low confidence identified. The laboratories reporting the additional variants stated they would have needed more DNA for further testing to confirm. As these variants were not reproduced in other laboratories or by other methods, they were highly likely to be introduced errors.

To determine why the known variants were not identified, the expected results were reported back to the participating laboratories to allow them to re‐evaluate their results, and a secondary data analysis was performed on all participants’ returned BAM files, apart from P8 who were unable to return sequence level data. The data re‐evaluation is summarized in Table 3.

Fifteen variants out of the 25 not reported in the initial evaluation were found to be present in the data on re‐analysis either by the participants themselves or the secondary analysis, which resulted in a concordance of 89% where samples passed the individual laboratories’ QC criteria.

Only three expected variants were not detected on re‐analysis. The reasons for non‐detection of these were as follows:

1. The sample containing the large insertion (*BRCA*1 exon13ins6kb) was not detected using any method in the primary analysis. The sequence level analyses undertaken were not developed to detect this form of *BRCA* variant so this was not an unexpected outcome. Only participant P7 claimed to detect this form of variant, but was not able to test this sample by MLPA‐NGS due to insufficient input DNA remaining following the TruSeq panel. However, P9, using a hybrid capture technique, found evidence of this variant on subsequent inspection of the data. See section on copy number analysis for additional information.
2. The *BRCA2* c.7788delAinsGGGT p.(Gly2596dup) VUS in sample 9 was not detected using the TSCA 2‐gene HRD panel used by P5. This variant was located at the 3′ and 5′ end, respectively, of two large (>160 bp) overlapping amplicons and the sequencing was performed using only 150 bp paired‐end reads, therefore in both instances, the region containing the variant was only covered by one read but not its counterpart paired read. This situation led to the variants being identified with a “strand bias” flag, and therefore eliminated from the filtered list of good‐quality variants.
3. Sample 12, an admixture containing a *BRCA2* c.10024G > A p.(Glu3342Lys) VUS and the lowest DNA concentration sample (66 ng/μL), was not detected using the TruRisk gene panel used by P9, probably due to sub‐optimal DNA quantity being used for this assay.

The primary reason for the initial non‐concordance was due to downstream results processing and/or interpretation. Specifically these reasons were:

Sample 5 that contained an intronic c.213‐11T > G pathogenic variant in *BRCA1* was missed using four laboratories’ data analysis methods as the pipelines used did not analyze beyond ±2 bp of the canonical splice site into the *BRCA1/2* intronic sequences.
Sample 12, an admixture sample, containing the *BRCA2* c.10024G > A p.(Glu3342Lys) was not reported by P5 and P8 as these laboratories considered it a benign variant. The classification of this variant caused disagreement among investigators as to whether it was a VUS or benign variant. It was designated benign in the ClinVar database (Landrum et al., 2016), but classified as a VUS through a more systematic investigation by other laboratories provided the sample passed QC.

The pathogenic *BRCA2* variant (c.6952C > T p.(Arg2318Ter) present at a low level in Sample 11 was not called using two data analysis processes as it was below the 10% allele frequency cut‐off set for these assays to avoid false positive miscalling of artifacts. The variants were present in the data at allele frequencies of 3% and 5% using these methods.

The automated data analysis process developed by P10 caused a number of problems resulting in the use of the wrong reference sequence and HGVS nomenclature, thus causing issues with detection and classification of variants. All the variants were detected using a secondary analysis method, and also by the originating laboratory after resolving their initial analysis issues.

In summary, from Table 3, we can conclude that after re‐evaluation of the data the amplicon‐based methods detected 67 of 75 (89%) assessable variants, whereas hybrid capture detected 21 of 23 (91%). When the 6 kb insertion is discounted as neither approach was designed or claimed to detect large genomic changes, they detected 67/68 (98.5%) and 21/21(100%), respectively, with only one laboratory missing one variant (a 3 bp indel) meaning that the two methods are substantially equivalent. The major source of discordance, however, was the bioinformatic pipeline and variant annotation. Overall, the specificity of variant calling was 100% for all participants as no false positives were reported in the end. The sensitivity ranged from 0% (zero true positive variants out of eight) to 87.5% (seven true positive variants out of eight) per laboratory.

#### 3.3.1 VAF comparison

**Figure 2:**
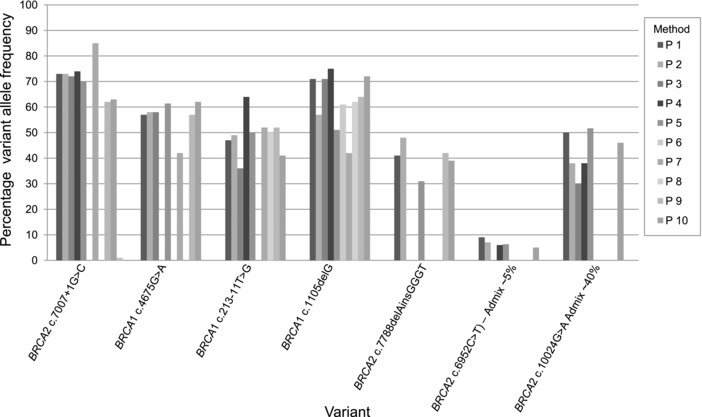
Comparison of variant allele frequencies across samples and processes. No trend can be observed between the laboratories (P1–P10)‐reported variant allele frequencies

The tumor variant allele frequencies of the variants were compared across the methods (Figure 2). No consistent trends were observed.

### 3.4 Central bioinformatic analysis of the sequencing data

#### 3.4.1 QC

To QC the sequence level data, the base qualities were plotted using FastQC. The general trend seen across all the samples is visualized for one of the samples sequenced by all laboratories in Supp. Figure S2b. A systematic downward shift in base qualities was observed in the data from laboratory P9 (Supp. Figure S2c). This did not transfer to noisy variant calls but required tuning down the base quality filters in variant calling. In the data provided by P2, the bioinformatics partner of the laboratory fused some of the reads at the raw data level, causing read lengths longer than the original read length and spurious changes in the QC plot towards the tails of the reads (Supp. Figure S2a).

#### 3.4.2 Sequencing coverage

**Figure 3:**
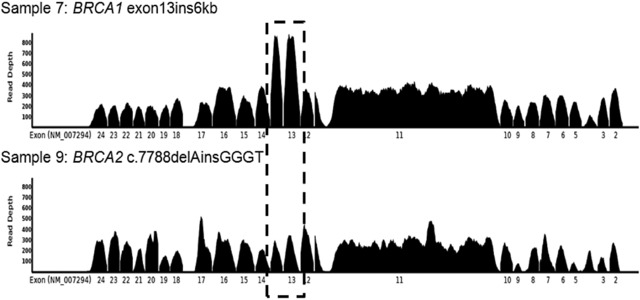
Evidence of copy number change in *BRCA1* at exon 13 in sample 7, a known carrier of a *BRCA1* exon 13 insertion. The boxed region highlights the amplified region in *BRCA1* exon 13. No clear copy number change was present for either *BRCA1* or *BRCA2* in any other sample evaluated in this way. Sample 9 is included as a typical comparator

Coverage analysis of the samples across the laboratories revealed certain regions not covered by all the panels. This is highlighted for *BRCA1* exon 19 (chr17:41203080–41203136), laboratory P7 in Supp. Figure S3. This exon has multiple pathogenic variants according to ClinVar that if missed would lead to false negatives. A consistent drop off in the GeneRead panel data across laboratories P1–P3 is shown in *BRCA2* around the region chr13:32930565–32930590 in Supp. Figure S4. The region also has clinically significant variants in ClinVar. In this study, no variants were missed due to these drop outs. The very high coverage in the amplicon‐based approaches was expected as PCR duplicates could not be marked. For the hybridizations capture approaches, very uniform coverage was observed (Figure 3; laboratories P9 and P11). The depth of coverage was on average sufficient for variant calling at 5% allelic fraction.

#### 3.4.3 Small variant analysis

All the provided data were run through central variant calling using VarDict. Identical variant calling settings were applied except for lower base quality filters for laboratory P9 whose data had a systematic downward base quality shift. The number of variants prior to filtering using any clinical actionability criteria was observed for each laboratory. This provided an overview of background noise in the panels. The number of variants called at 5% and 20% allele fraction thresholds are provided in Supp. Table S1 and S2. The short amplicon and hybrid capture protocols showed the least spurious calls prior to downstream filtering.

#### 3.4.4 Copy number analysis

Large genomic insertion, inversion, and deletion variants represent a significant class of pathogenic germline variants (Ewald et al., 2009). Although the majority of the methods here, including the bioinformatics processes, were not tuned to detect structural variation, the data from sample 7 (*BRCA1* exon13ins6kb) was analyzed post‐hoc to determine if a robust copy number increase in exon 13 could be called.

Where data were available, BAM files were analyzed using Seq2C for both *BRCA* genes. Log2 values, normalized to the sample median, were plotted for each exon. Using this approach however, the robust detection of this variant proved difficult. This was possibly due to there being no control in the sample subset and the cohort size used for normalization being smaller than the ideal number of >30. However, visual examination of the coverage data by P9 using the TruRisk Sureselect XT (Agilent) 48 gene panel revealed an amplification in the region encompassing *BRCA1* exon13 (Figure 3). There was also evidence of this variant seen in the hybridization capture‐based reference data (see Supp. Figure S1). However, as Supp. Figure S5 shows, in amplicon capture‐based data, it is not evident which exon is amplified as the copy number profile is not the highest for the actually amplified exon.

## 4 DISCUSSION

*BRCA1/2* testing in FFPE tissue samples is a multistep process involving pathology review, DNA extraction and quantification, an enrichment methodology, library preparation, generation of sequencing data, bioinformatic analysis, and detection of variants as well as variant classification to determine the implications for the patient. This unsurprisingly results in a diverse range of *BRCA1/2* testing options available to screen for *BRCA1* and 2 germline and somatic variants in DNA extracted from FFPE tissue.

The laboratories in this study selected their testing methods for a number of different reasons. Laboratories using amplicons‐based approaches believed these methods were more robust with low quality and quantity DNA samples. Some laboratories used commercial kits because no assay development was required, they expected the assay to be validated, especially for European Conformity‐in vitro diagnostics, and specifically designed for use on FFPE DNA. Certain laboratories chose to use multi gene panels, including those laboratories using hybrid capture, as they were more universal for the analysis of other samples and including other genes in the homologous recombination repair pathway that could help future‐proof the assays. Some laboratories selected methods that could be developed for both blood and tumor testing so only one workflow had to be established. The TSCA dual strand protocol and the smMIP‐based enrichment methods were used as they allow strand specific targeting, thus distinguishing any artifacts from bona‐fide variants in FPPE material. smMIPs have the additional advantage of a unique molecule tag rendering differentiation between reads derived from PCR duplicates and those from independent DNA molecules possible. Some systems, such as the TruRisk gene panel were considered to be very flexible allowing additional genes to be analyzed as required. Having previous experience and expertise with similar methods were also key selection criteria.

This study was designed to compare and contrast different methodologies and to highlight the advantages and disadvantages of different approaches. It was not a proficiency study to test the laboratories’ competencies. As such, a challenging set of DNA sample were supplied to allow the methods to be evaluated over a range of conditions such as DNA amount and probably resulted in a higher failure and discrepancy rate than might be expected. Ninety‐eight analyses generated sequencing data out of a maximum of 117 with nine laboratories analyzing a full cohort of 12 samples and one laboratory using only nine samples. Of these 117 possible results, 19 analyses failed (16%), see Table 3. Eighty‐two analyses were reported to contain the expected genotype (70%) and 16 analyses (14%) did not have the expected genotype reported. No sample was failed by all laboratories.

It is useful to determine how methods perform over a range of DNA inputs so that the best method for a laboratory's anticipated samples can be selected. However, in a study of this kind, even if a method does not perform over a wide range of DNA amounts, it may still perform well using the laboratories’ own processes using samples from their own institutions, processed and quantified in an optimal way for downstream testing. Failures were mainly clustered using two approaches, P6 and P7 (*n* = 13, 11%), see Table 3. The method used by P7 required a higher quality or quantity of DNA than supplied for use in their 30 gene amplicon and 12 gene MLPA‐NGS panels to achieve reproducible results from FFPE samples. P6 failed at both higher and lower DNA input. The required input was 500 ng (Table 2) measured by nanodrop and this may not have equated to the DNA amount supplied (measured by quantitative PCR). The remaining fails were either due to input DNA being too low (*n* = 1), input DNA being too high due to inaccuracy of DNA measurement (*n* = 1) or unknown reasons (*n* = 4). No sample‐specific failure pattern was observed with the most failures (three laboratories) being in samples 4, 9, and 11. Samples 5 and 6 were not failed by any laboratory.

Data analysis was the main reason for not reporting variants that were found present in the sequencing data. On re‐analysis using a second bioinformatics process and/or re‐analysis by the laboratory generating the data, all but 11 of the variants were detected, giving a concordance of 89% for samples that passed sequencing quality metric criteria. Analysis parameters and settings varied between processes implemented across the laboratories. For example, the intronic variant *BRCA1* c.213‐11T > G, was not reported by four laboratories as it was greater than 2 bp into the intron and thus beyond the canonical splice sites where the laboratories or the bioinformatics programmes set analysis cut‐offs. The databases and data sources used to assist with *BRCA1/2* classification also influenced the classification and subsequent reporting of variants. The *BRCA2* c.10024G > A variant, considered by most laboratories as a VUS, was classified as benign in the ClinVar database (Landrum et al., 2016) and also by some laboratories. This disagreement highlights an issue with the classification of certain *BRCA* variants as even experienced teams can differ in their opinion as to the classification. However in the case of this variant, a patient with either classification would not have been eligible for treatment with PARP inhibitors. The VAF cut‐offs used to detect low‐level variants also varied between laboratories and samples with clinically relevant variants present at a low level were not reported by three participating laboratories as the threshold for reporting was not reached. Despite the possibility of false negative results, the precise level of cut‐off should be determined during analytical validation of the assay as the risk of false positive calls increases with lower level cut‐offs. The risk of missing a low‐level variant below the reporting cut‐off can be mitigated by ensuring that samples are adequately reviewed by a pathologist prior to testing and the neoplastic cell content estimated as adequate for the analysis. Obviously in this study, this review was not possible as the participants received DNA. These analysis issues highlight the importance of validation of the data analysis pipelines for the complex analysis of the *BRCA1/2* genes.

Central bioinformatic analysis of all data sets together revealed that for example lower base qualities in sequencing did not correlate with a higher number spurious variant calls (P9 in Supp. Table S1). The two can therefore be seen as orthogonal metrics of quality. Generally the higher rates of spurious calls were seen in data from the longer amplicon sequencing approaches. Low background noise levels were seen especially in the hybridization capture‐based data. While downstream filtering and actionability criteria meant that no false positives were reported, approaches that produce fewer spurious candidate variants are preferred when expanding variant calling to lower and lower allele fractions. While coverage was sufficient on average in all panels, some panels had drop outs for some bases. It is crucial to check minimum coverage is sufficient for all bases to prevent false negatives.

In this study, the majority of methods used were not developed or optimized for the detection of copy number variants, hence the reason for the non‐detection for nine cases of discordance. Nevertheless, one laboratory using a hybrid capture‐based approach observed an increase in copy number in the expected locus for the sample containing a *BRCA1* exon13ins6kb in a retrospective analysis with knowledge of the variant and the positive sample. The same region of amplification was also observed with knowledge of the variant in the reference laboratory data. Another laboratory used a customized MLPA‐NGS assay, which had been shown to detect germline LGRs in FFPE tumor samples (Kondrashova et al., 2015); however, there was insufficient DNA provided to permit analysis of this sample. The detection of this variant with confidence in the data generated using the other evaluated methods proved difficult. A larger study to explore methods able to detect LGRs in FFPE is warranted. In general, the hybridization capture‐based methods provided far smoother coverage and copy number profiles compared with the amplicons‐based approaches due primarily to the ability to remove PCR duplicates in hybridization capture data.

There were limitations to this study. Only the capture, sequencing, and variant calling steps were evaluated and all participants relied on Illumina sequencing technology only. The primary aim was to focus on the analytical process for comparison, hence every participant received exactly the same DNA samples for analysis. This allowed better comparison of the methods without confounding factors such as sample processing that would have been likely to introduce more variation. However this did introduce unforeseen problems: some participants re‐quantified the DNA received to determine how much to add to the tests, whereas others did not due to the limited amount of DNA supplied. In at least one laboratory (P4), this resulted in too much DNA being added resulting in increased test failure. This highlights the issue that different DNA measuring methods do not generate the same result and any laboratory receiving DNA only should still measure the DNA sample using the method their assay was optimized with.

## 5 CONCLUSIONS

The NGS approaches used in this study were able to detect the *BRCA1/2* variants in this diverse sample well when optimal DNA amounts were used and once data analysis issues were resolved. Comparing the initial versus re‐evaluation data eight laboratories using amplicon‐based methods missed seven additional variants, whereas two laboratories using hybrid capture missed eight additional variants, seven by one laboratory alone. This highlights that the bioinformatic analysis and annotation were much more responsible for discordance than the detection method. The overwhelming source of error was therefore human interpretation. This is the major message of this manuscript and emphasizes the need for better standards, given the impact this could have clinically. As much consideration should be given to validating the data analysis and interpretation processes as to the “wet laboratory” NGS process. The bioinformatic analyses revealed the need for better standardization of *BRCA* variant notation and classification, such as *BRCA* Exchange (https://brcaexchange.org/). Extending variant calling into intronic regions beyond the splice sites was also found to be crucial. Based on the results of this study, it is recommended to use hybridization capture‐based technology for DNA targeting especially if LGRs or accurate copy number profiles are required. If cost or DNA input rules out the use of hybridization capture, short PCR amplicon capture can provide excellent quality data for small variant calling. Longer amplicon kits suitable for germline sequencing tend to lead to high levels of noise and poor capture of the fragmented DNA in FFPE.

Given all these considerations, it is important to drive standards and standardization in *BRCA* FFPE testing particularly when the test results dictate clinical decisions regarding life extending therapies.

## Supporting information

Supp. Figure S1: Copy number profile across BRCA1 exons for sample S07 (reference laboratory).
Further evidence of copy number changes in BRCA1 at exon 13 in sample 7 in the reference data analysed using Seq2C [20] with Log 2 values normalised by sample median. The boxed region indicates the location of the amplification.
Supp. Figure S2: Base quality profiles for sample S01 across three participants (laboratories 2 (a), 3 (b) and 9(c)) to highlight systematic differences. The x‐axis reflects the sequencer cycles (up to read length) and y‐axis the Phred base quality
Supp. Figure S3: BRCA1 coverage profiles for the 12 samples for four participants highlighting differences between amplicon (top row) and hybrid capture approaches (bottom row). Exon 19 drop out in laboratory P7 data can be observed across the samples.
Supp. Figure S4: BRCA2 coverage profile drop outs in GeneRead amplicon panel data in chromosome 15.
Supp. Figure S5: Copy number profile for an amplicon capture, sample S07.
Table S1: Variant calls per sample by central bioinformatic analysis prior to BRCA specific filtering using 5% as the allele fraction cutoff. Green colouring is used to indicate one correct detected variant. Red colouring represents discordant results.
Table S2: Variant calls per sample by central bioinformatic analysis prior to BRCA specific filtering using 20% as the allele fraction cutoff. Green colouring is used to indicate one correct detected variant. Red colouring represents discordant results.
