# Supplementary material for: An evaluation of the challenges to developing tumor BRCA1 and BRCA2 testing methodologies for clinical practice
Source: Hum Mutat. 2017 Dec 28;39(3):394–405. doi: 10.1002/humu.23375 (PMC5838520; doi:10.1002/humu.23375)
Supplement: Supplementary file 1 — Supp. Figure S1: Copy number profile across BRCA1 exons for sample S07 (reference laboratory). Further evidence of copy number changes in BRCA1 at exon 13 in sample 7 in the reference data analysed using Seq2C [20] with Log 2 values normalised by sample median. The boxed region indicates the location of the amplification. Supp. Figure S2: Base quality profiles for sample S01 across three participants (laboratories 2 (a), 3 (b) and 9(c)) to highlight systematic differences. The x‐axis reflects the sequencer cycles (up to read length) and y‐axis the Phred base quality Supp. Figure S3: BRCA1 coverage profiles for the 12 samples for four participants highlighting differences between amplicon (top row) and hybrid capture approaches (bottom row). Exon 19 drop out in laboratory P7 data can be observed across the samples. Supp. Figure S4: BRCA2 coverage profile drop outs in GeneRead amplicon panel data in chromosome 15. Supp. Figure S5: Copy number profile for an amplicon capture, sample S07. Table S1: Variant calls per sample by central bioinformatic analysis prior to BRCA specific filtering using 5% as the allele fraction cutoff. Green colouring is used to indicate one correct detected variant. Red colouring represents discordant results. Table S2: Variant calls per sample by central bioinformatic analysis prior to BRCA specific filtering using 20% as the allele fraction cutoff. Green colouring is used to indicate one correct detected variant. Red colouring represents discordant results. [file HUMU-39-394-s001.pdf]

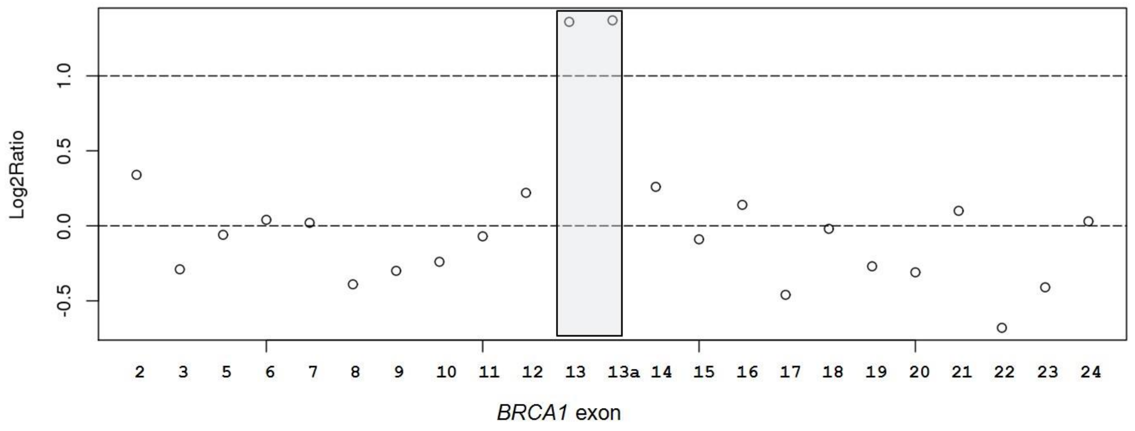

Supp. Figure S1: Copy number profile across *BRCA1* exons for sample S07 (reference laboratory).
Further evidence of copy number changes in *BRCA1* at exon 13 in sample 7 in the reference data analysed using Seq2C [20] with Log 2 values normalised by sample median. The boxed region indicates the location of the amplification.

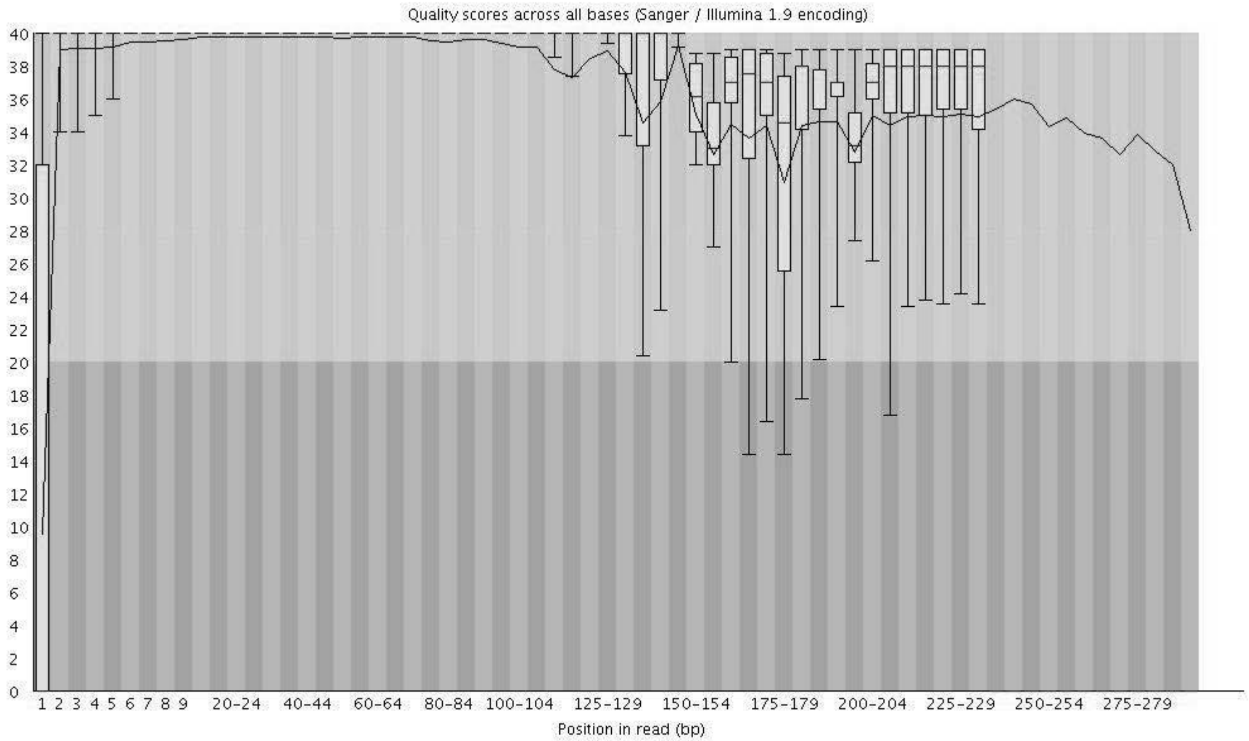
Quality scores across all bases (Sanger / Illumina 1.9 encoding)
0 2 4 6 8 10 12 14 16 18 20 22 24 26 28 30 32 34 36 38 40
1 2 3 4 5 6 7 8 9 20-24 40-44 60-64 80-84 100-104 125-129 150-154 175-179 200-204 225-229 250-254 275-279
Position in read (bp)

a)

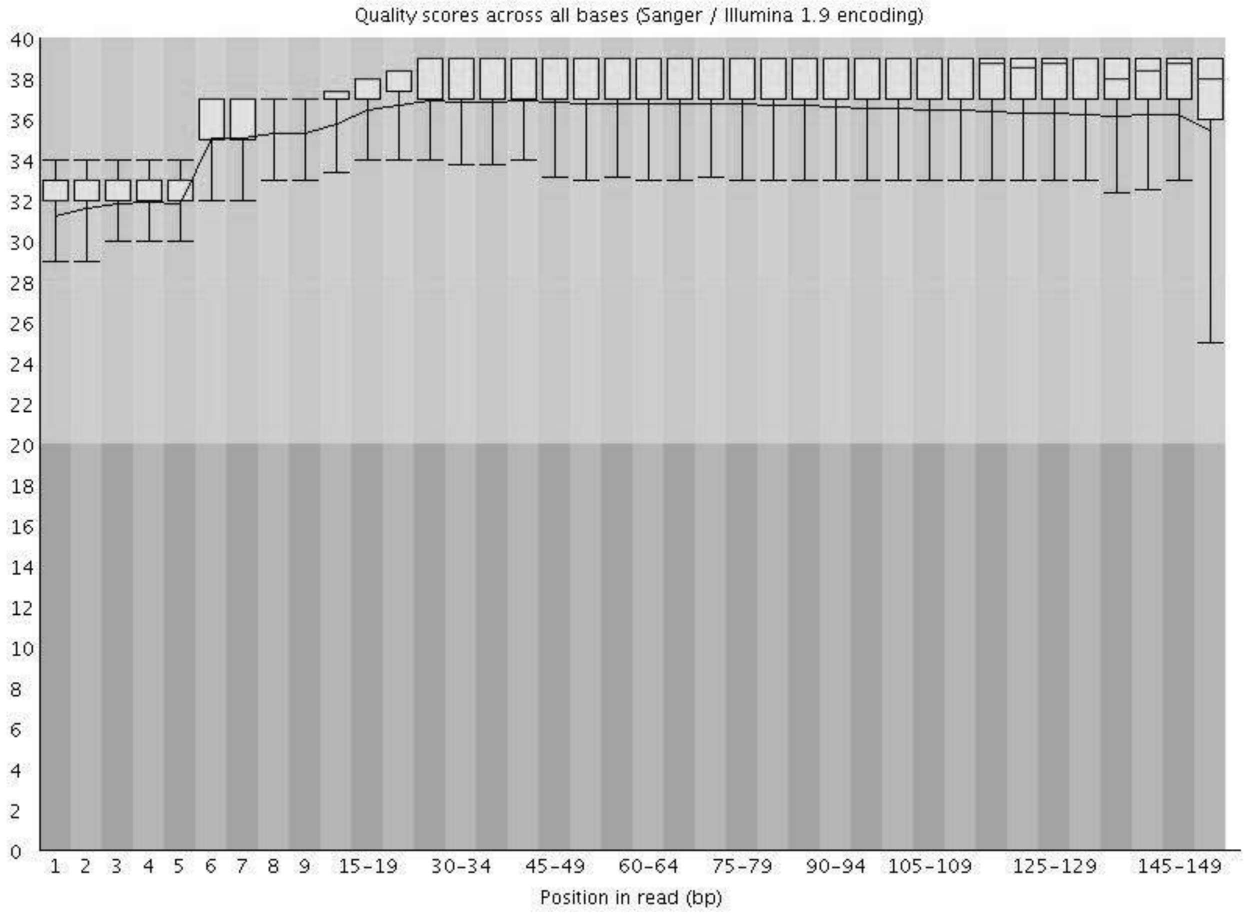
Quality scores across all bases (Sanger / Illumina 1.9 encoding)
40
38
36
34
32
30
28
26
24
22
20
18
16
14
12
10
8
6
4
2
0
1 2 3 4 5 6 7 8 9 15-19 30-34 45-49 60-64 75-79 90-94 105-109 125-129 145-149
Position in read (bp)

b)

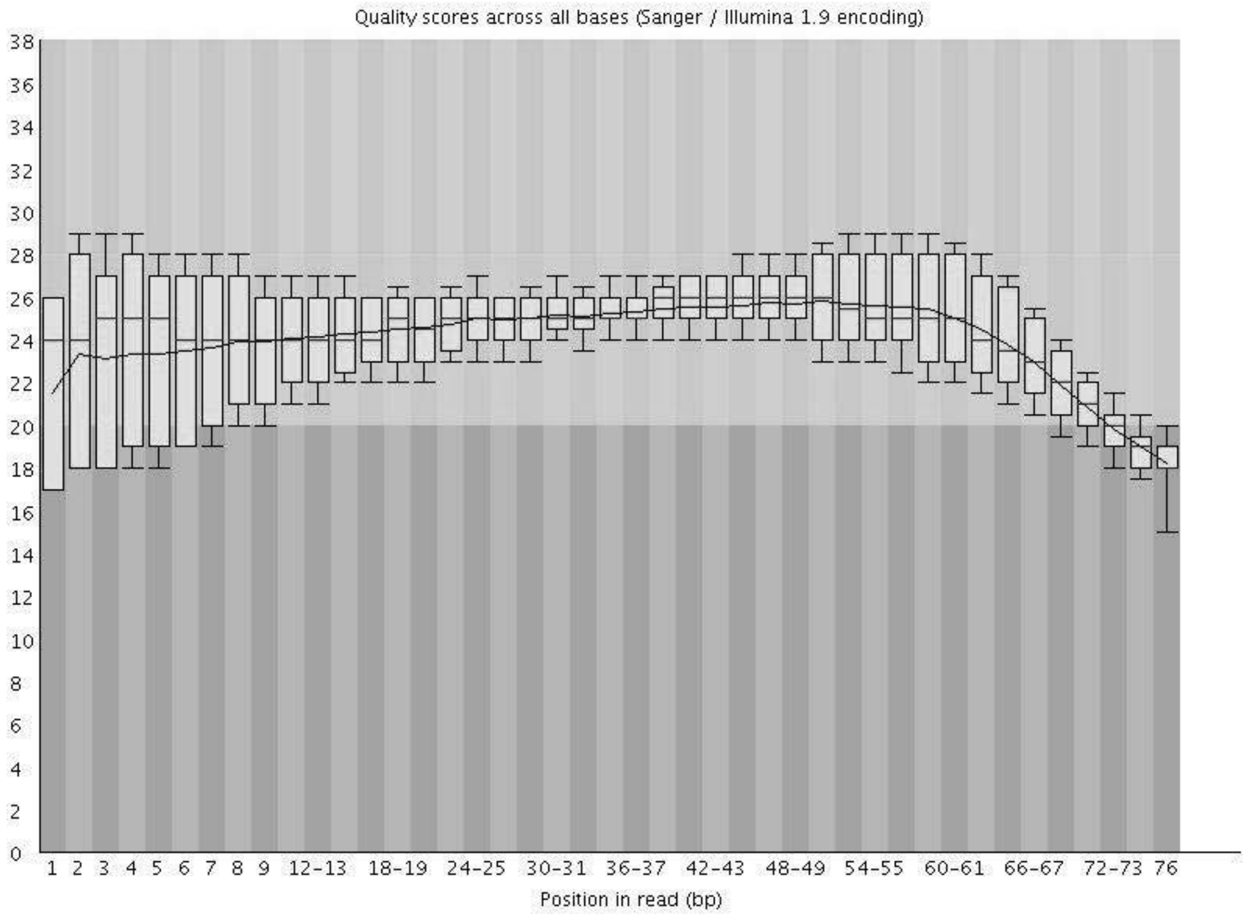

c)

Supp. Figure S2: Base quality profiles for sample S01 across three participants (labs 2 (a), 3 (b) and 9(c)) to highlight systematic differences. The x-axis reflects the sequencer cycles (up to read length) and y-axis the Phred base quality.

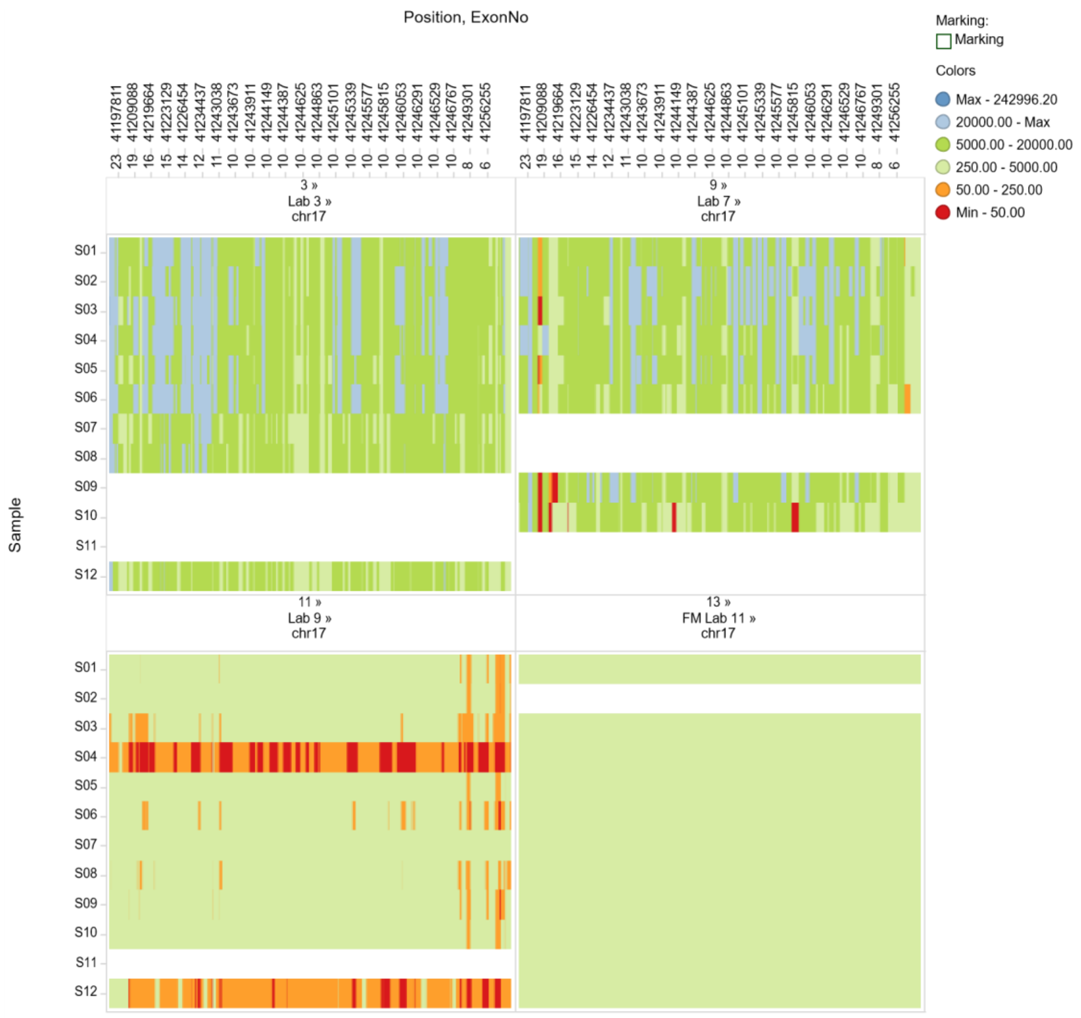

Supp. Figure S3: *BRCA1* coverage profiles for the 12 samples for four participants highlighting differences between amplicon (top row) and hybrid capture approaches (bottom row). Exon 19 drop out in lab P7 data can be observed across the samples.

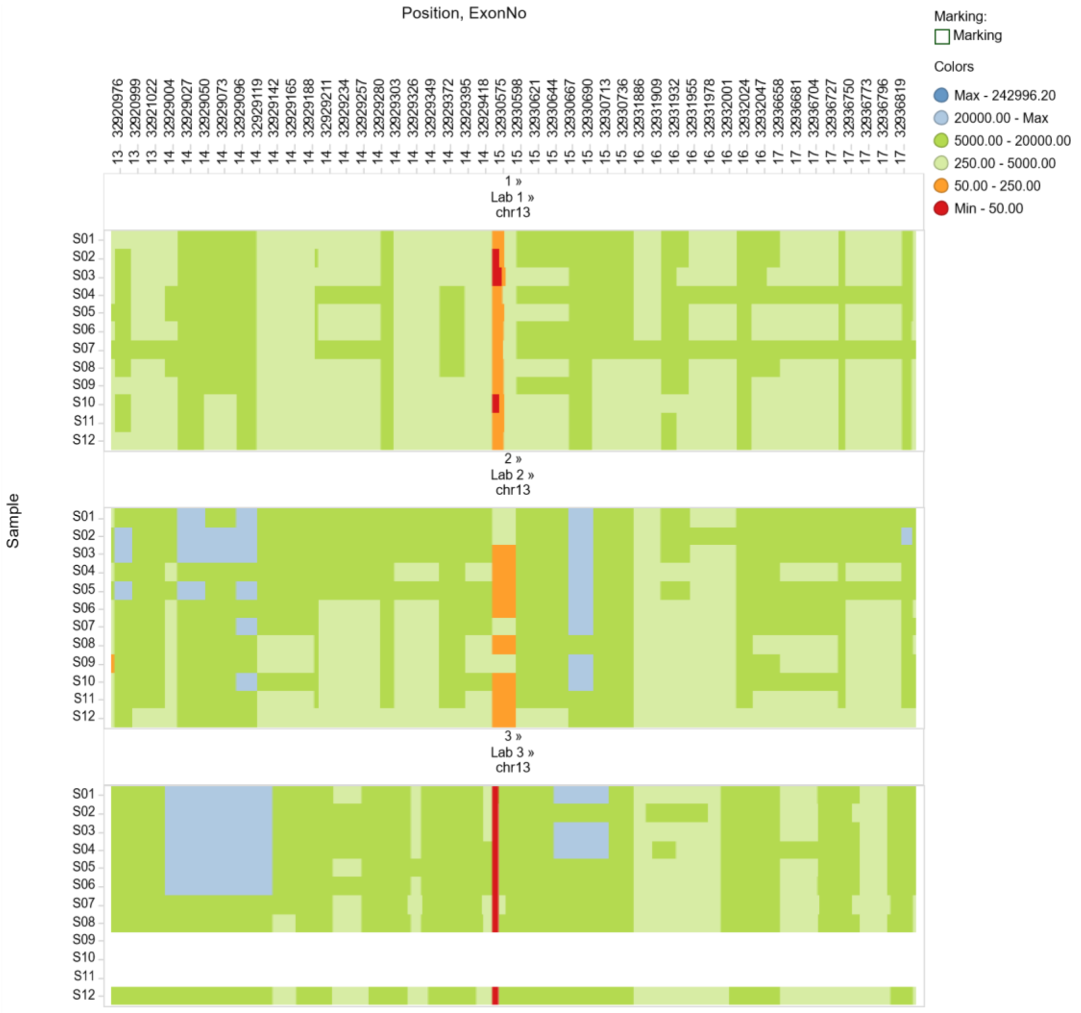

Supp. Figure S4: *BRCA2* coverage profile drop outs in GeneRead amplicon panel data in chromosome 15.

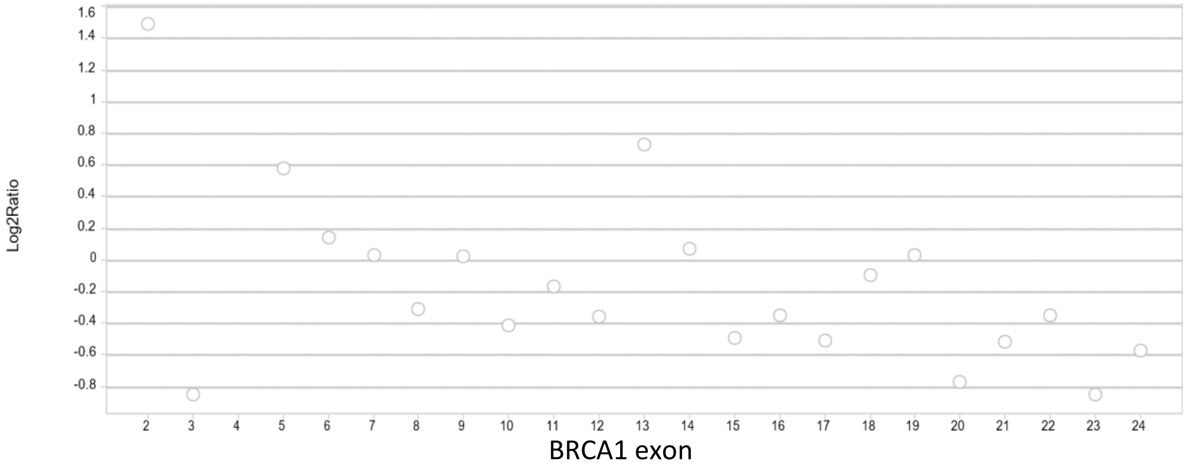

Supp. Figure S5: Copy number profile for an amplicon capture, sample S07.

Table S1: Variant calls per sample by central bioinformatic analysis prior to BRCA specific filtering using 5% as the allele fraction cutoff. Green colouring is used to indicate one correct detected variant. Red colouring represents discordant results.

	Lab 1	Lab 2	Lab 3	Lab 4	Lab 8	Lab 8	Lab 5	Lab 5	Lab 6	Lab 7	Lab 9	Lab 10	Lab 11
S01	1	1	1	1	3	1				37	1	2	1
S02					2								
S03				1	2	2				34			
S04	1	1	1		2	3				67		1	1
S05	1	1	1	1	2	3				31	1	1	1
S06		1	1	1	2	5	2	1		125	1	4	1
S07				1	63	60		1				1	
S08					105	112						1	
S09	1	1			12	13				225		2	1
S10					14	15				203		2	
S11	1	1		1	2	3	2	2				1	
S12	1	1	1	1	82	87							1

Table S2: Variant calls per sample by central bioinformatic analysis prior to BRCA specific filtering using 20% as the allele fraction cutoff. Green colouring is used to indicate one correct detected variant. Red colouring represents discordant results.

	Lab 1	Lab 2	Lab 3	Lab 4	Lab 8	Lab 8	Lab 5	Lab 5	Lab 6	Lab 7	Lab 9	Lab 10	Lab 11
S01	1	1	1	1	1					1	1	1	1
S02													
S03													
S04	1	1	1		1	1				10		1	1
S05	1	1	1	1	1	1				11	1	1	1
S06		1	1	1	1	1	1	1		5	1	1	1
S07					9								
S08					5	5							
S09	1	1				1				6		1	1
S10					1					47			
S11													
S12	1	1	1	1	3	2							1
